# Supplementary material for: Splice-Junction-Based Mapping of Alternative Isoforms in the Human Proteome
Source: Cell Rep. Author manuscript; Available in PMC 2020 Jan 15. (PMC6961840; doi:10.1016/j.celrep.2019.11.026)
Supplement: 2 [file NIHMS1546469-supplement-2.zip › DF1/PXD010154/Spleen/Spleen_18_MKRN1_LEYSGMISAHHNLCLLGSSDSPTSASRVVGITDMNTATTSGAGAAAA.pdf]

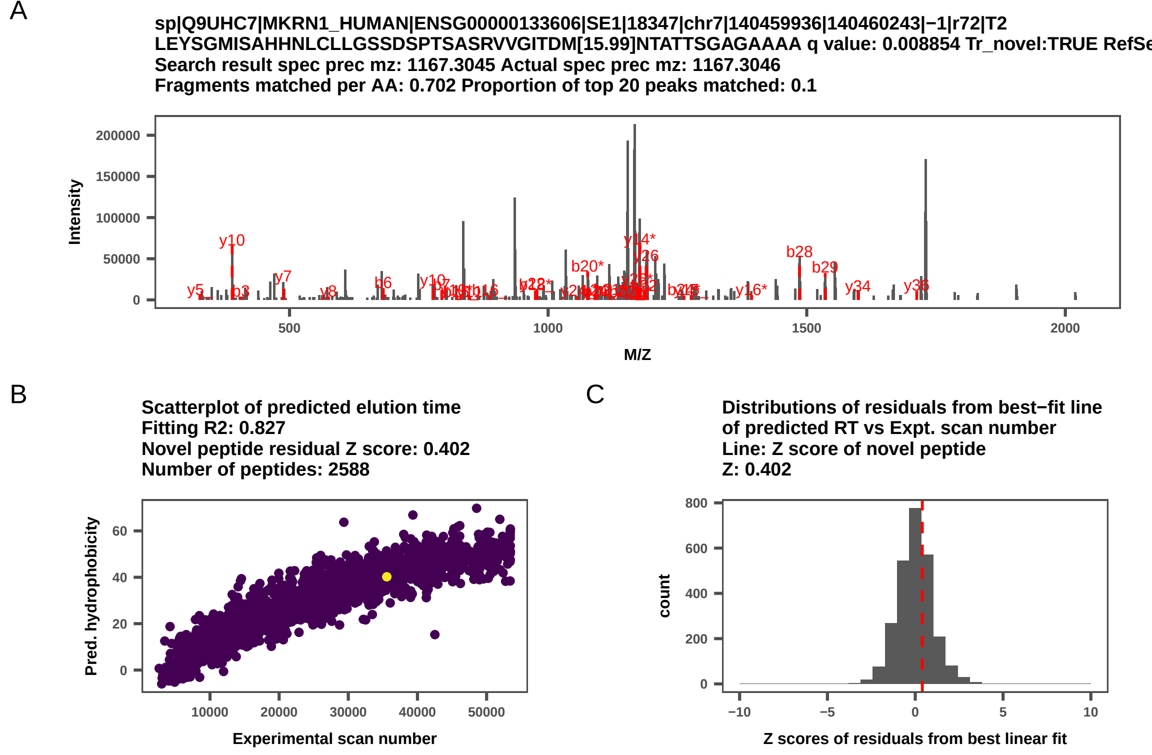
A
sp|Q9UHC7|MKRN1_HUMAN|ENSG00000133606|SE1|18347|chr7|140459936|140460243|−1|r72|T2
LEYSGMISAHHNLCLLGSSDSPTSASRVVGITDM[15.99]NTATTSGAGAAAA q value: 0.008854 Tr_novel:TRUE RefSe
Search result spec prec mz: 1167.3045 Actual spec prec mz: 1167.3046
Fragments matched per AA: 0.702 Proportion of top 20 peaks matched: 0.1
Intensity
200000
150000
100000
50000
0
y10
y14*
y26
b28
b20*
b29
y7
b6
y5
b3
y8
y34
y36
y16*
500
1000
1500
2000
M/Z
B
Scatterplot of predicted elution time
Fitting R2: 0.827
Novel peptide residual Z score: 0.402
Number of peptides: 2588
Pred. hydrophobicity
60
40
20
0
10000
20000
30000
40000
50000
Experimental scan number
C
Distributions of residuals from best−fit line
of predicted RT vs Expt. scan number
Line: Z score of novel peptide
Z: 0.402
count
800
600
400
200
0
−10
−5
0
5
10
Z scores of residuals from best linear fit
